# Alternating-magnetic-field induced enhancement of diffusivity in Ni-Cr alloys

**DOI:** 10.1038/s41598-017-18500-w

**Published:** 2017-12-22

**Authors:** Chuanjun Li, Shengya He, Hannes Engelhardt, Tongjun Zhan, Weidong Xuan, Xi Li, Yunbo Zhong, Zhongming Ren, Markus Rettenmayr

**Affiliations:** 1 State Key Laboratory of Advanced Special Steel & Shanghai Key Laboratory of Advanced Ferrometallurgy & School of Materials Science and Engineering, Shanghai University, Shanghai, 200072 China; 2 Otto Schott Institute of Materials Research, Friedrich-Schiller-Universität Jena, 07743 Jena, Germany

## Abstract

For applying an alternating magnetic field (AMF) in materials processing it is of high significance to understand the physical mechanisms behind the change in diffusivity in the AMF. In this work, the effect of the AMF on interdiffusion in a Ni-Cr alloy was investigated with a diffusion couple. The interdiffusion coefficient was found to increase with increasing AMF intensity. The faster diffusivity is a consequence of the enhancement of the dislocation density in the diffusion couples that was confirmed by the broadening of X-ray diffraction peaks. The higher dislocation density is attributed to the magnetoplastic effect (MPE). Theoretical considerations on the relation of MPE, dislocation density and diffusivity are in agreement with the experimental results.

## Introduction

Diffusion plays a crucial role in numerous kinetic processes in alloys, e.g. homogenization, precipitation, creep and corrosion. From the practical point of view, it is desirable to influence diffusion rates of the alloying elements. For example, the rate of Cr diffusion into Ni essentially determines the maximum thickness of commercially thoriated Ni-Cr alloys and thus the economy of their manufacturing^1^. The addition of rare earth elements to high temperature alloys can improve their oxidation resistance and reduce the growth rate of oxides^2^. Furthermore, an increase in diffusivity could be exploited to shorten the time for heat treatment of a variety of alloys. Thus, influencing the diffusion rates of elements in alloys is of direct practical significance. Some methods are known to modify the diffusivity, e.g. adding alloying elements^3^ and applying external fields such as magnetic^4–6^ or electric fields^7^.

Over the past decades, a magnetic field has extensively applied to materials processing^8^ and various novel magnetic phenomena have been found^9,10^. Alternating magnetic fields (AMFs) as an effective method for modifying structures and properties of materials have attracted considerable attention. It has been found that AMFs refine grains^11^, reduce macro/micro segregation^12,13^, accelerate stress release^14^ and so forth. At present, AMFs are widely applied in industrial production processes such as electromagnetic continuous casting, induction heating and levitation. Over the past decade, it has been confirmed that an AMF has the capability of changing the diffusion rate in different alloys, e.g. steel^15^, Al-Mg, Al-Zn, Al-Cu^4,16^ and Mg-Gd^17^. Nevertheless, the mechanisms behind the change in diffusivity in an AMF are still not fully clear. Although the ambipolar diffusion theory was proposed to interpret diffusion inhibition in the alloy systems in a steady magnetic field^18^, it seems to be little success^19^. Moreover, the theory is not applicable to the case in AMF. Thus, the objective of the present work is to i) experimentally examine the change in diffusivity in a Ni/Ni-10wt%Cr diffusion couple in the presence of an AMF and ii) to quantitatively account for the diffusion phenomena in the AMF.

## Results

**Figure 1:**
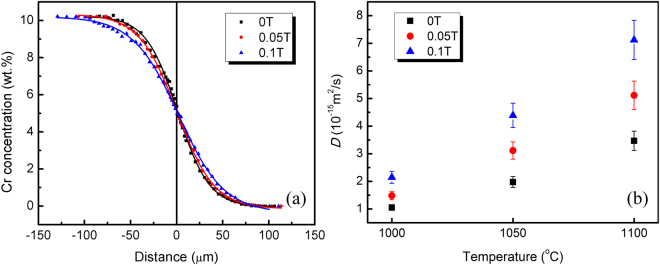
Concentration profiles in diffusion couples annealed for 32 h at 1100 °C (**a**) and interdiffusion coefficients for different AMF intensities (**b**). The origin x = 0 is the position of the Boltzmann-Matano plane.

Figure 1(a) shows Cr concentration vs. distance profiles generated in AMFs of 0 T, 0.05 T and 0.1 T at 1100 °C. It can be seen that the solute atoms diffuse distinctly faster in the AMFs. The same behavior was observed at 1000 °C and 1050 °C. In order to quantitatively compare the change in diffusivity, the diffusion coefficients were calculated from the concentration profiles using the Boltzmann-Matano method. Here, the interdiffusion coefficients at 5 wt%Cr concentration generated under different conditions are compared, as shown in Fig. 1(b). The diffusion coefficients in the samples that were annealed in an AMF of 0.05 T increased by 41% at 1000 °C, 58% at 1050 °C and 48% at 1100 °C, in comparison with those without an AMF. In the higher AMF of 0.1 T, the increase in the diffusion coefficient is more pronounced. It is concluded that an AMF significantly enhances the diffusivity of Cr in Ni and in Ni-Cr alloys.

**Figure 2:**
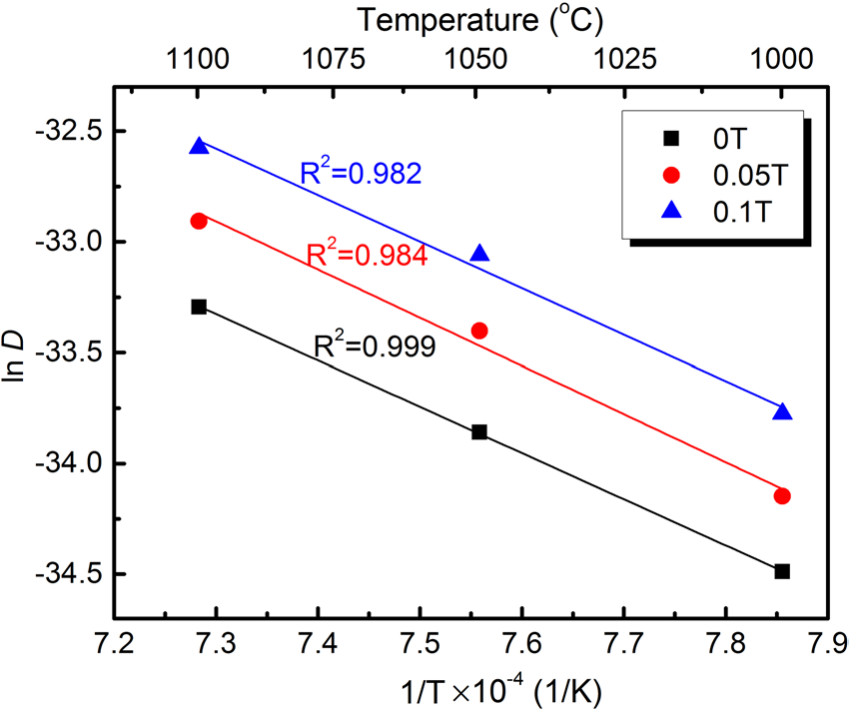
Logarithm of interdiffusion coefficients in the Ni-Cr alloy vs. reciprocal absolute temperature at different AMF intensities. The *R*-squared values for linear fitting also are given.

Figure 2 displays the logarithm of the interdiffusion coefficient versus the reciprocal value of the absolute temperature. From the fitting curves, we can extract the frequency factors and the activation energies for diffusion, as listed in Table 1. In the absence of an AMF, both parameters fluctuate considerably compared with data from references^20–22^, which may at least partly be attributed to the different preparation methods of the diffusion couples. However, those results still follow the general rule that the activation energies for lattice, dislocation and grain boundary diffusion decrease in this sequence. In this work, we employed a stainless steel holder to clamp the diffusion couple and artificially caused some visible plastic deformation of the diffusion couple in order to obtain a good diffusion interface. By the plastic deformation, the dislocation density was drastically increased, which is in turn expected to increase the average diffusion rate. Hence, the activation energy in this work is close to that in ref.^20^ for a fine grained material, but somewhat lower than the activation energies reported for bulk diffusion^21,22^.

**Table 1:** Comparison of frequency factors *D*_0_ and activation energies *Q* in this work and experimental data from literature.

AMF(T)	*D*_*0*_ (m^2^/s)	*Q*(kJ/mol)	Remark
0	1.4 × 10^−8^	173	This work
	3.0 × 10^−7^	192	ref.^20^
	9.4 × 10^−6^	266	ref.^21^
	5.2 × 10^−4^	289	ref.^22^
0.05	4.0 × 10^−8^	181	This work
0.1	3.2 × 10^−8^	174	This work

Considering that we consistently employed the same preparation method of all diffusion couples, we can compare the change in the diffusion coefficients with and without an AMF. From Table 1, the frequency factors increase by a factor of 2.8 and 2.3 in AMFs of 0.05 and 0.1 T, respectively. The activation energies also increase by 4.6% and 0.6% respectively. In the range of the experimental error, the change in the activation energies in the AMF is small enough to be negligible. AMFs thus mainly modify the frequency factor. This is in accordance with previous experimental observations in other alloy systems like Al-Zn^4^ and Mg-Gd^17^.

## Discussion

The present results show that AMFs exert a significant effect on the frequency factor, but only a small effect on the activation energy. The fact that the activation energy is essentially unaffected by the AMF is plausible from the following two aspects. On the one hand, for Ni and Ni-Cr alloys the magnetic free energy in an external field of 0.1 T is estimated to be in the order of 10^−8^ ~ 10^−9^ of the activation energy for diffusion at the given annealing temperatures, and thus it cannot be responsible for substantial changes. This is distinctly different from the case of the magnetic contribution to diffusivity in ferromagnetic alloys^23^. On the other hand, more fundamentally, the activation energy mainly consists of the electrostatic interaction energy between the diffusing impurity and the vacancy according to the diffusion theory by Lazarus^24^. Any change in the effective potential of the ion will influence the diffusion rate. However, it was shown that the effect of the magnetic field on the charge screening and thus the potential of the impurity ion is negligible in metals in a magnetic field less than 10^3^ T^18^. This underlines that a change in the activation energy in the magnetic field should not be expected.

In contrast, the frequency factor was distinctly modified by the AMFs. Although some investigators proposed that the change in the frequency factor in a magnetic field may be ascribed to the variation of the activation entropy, there is still a lack of experimental and theoretical evidence for this guess^4^. According to the atomic theory of diffusion, the frequency factor is expressed as *D*_0_ = (*a* ^2^/6) · *v* · exp(ΔS/*k*), where *a*, *v*, ΔS and *k* are the atomic jump width, the vibration frequency, the activation entropy and the Boltzmann constant, respectively^25^. Obviously, the change in vibration frequency and activation entropy in the vicinity of a vacancy can modify the frequency factor. Up to now, no attempt has been made to investigate the effect of an AMF on these variables to the authors’ knowledge.

The effect of the AMF on the vibration frequency is plausible, but unknown. The increase in the frequency factor in the present case can at least partly be attributed to the increase in the activation entropy owing to an increase of the dislocation density in the diffusion couple by the AMF, as demonstrated by XRD (see following section). It is well established that the mean jump frequency of atoms in dislocation cores is higher than that of the same atom in the lattice^26^, which leads to the higher diffusivity along dislocations compared with that by lattice diffusion. It is thus reasonable to attribute the larger part of the change in diffusivity in the AMF to the increase in the dislocation density.

**Figure 3:**
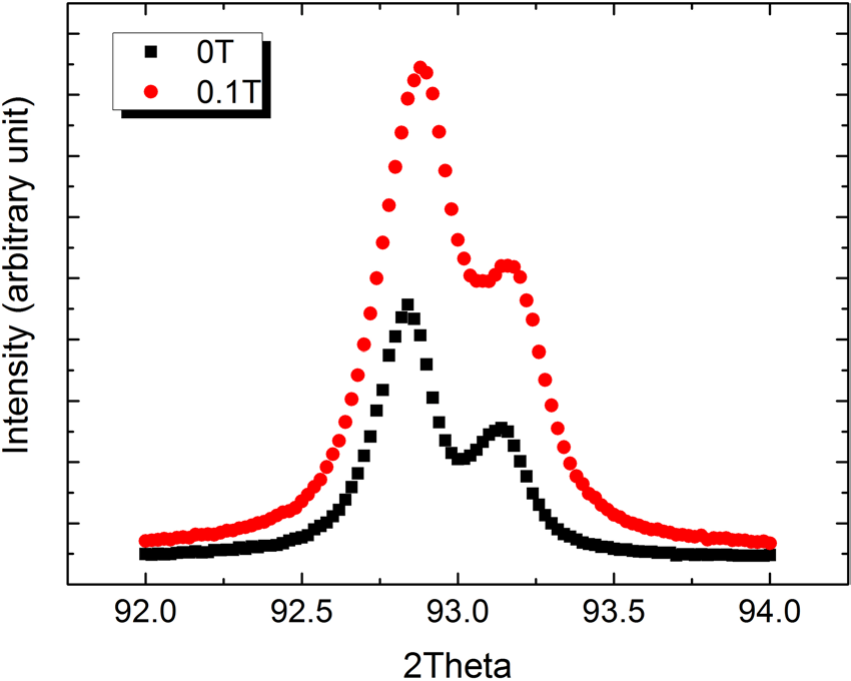
Diffraction peaks of the (311) reflection in diffusion couples annealed at 1100 °C with and without an AMF of 0.1 T.

In order to observe the difference in dislocation density in the diffusion couples after annealing with and without the AMF, XRD profiles of various reflections were examined. As an example, Fig. 3 displays two profiles of the (311) Bragg reflection with and without 0.1 T. It can be observed that the full width at half maximum (FWHM) in 0.1 T is broader than that without the AMF. Since the mean grain size, about 0.5 mm, in the diffusion couples under investigation is large, grain boundaries won’t play a role, and peak broadening can be a consequence of lattice defects such as dislocations. Further, the dislocation density can be calculated using Dunn’s equation^27^, ρ_d_ = (Δθ)^2^/(4.35*b* ^2^), where ρ_d_, Δθ and *b* are the dislocation density, the FWHM and the Burgers vector, respectively. For example, the calculated dislocation densities from the FWHM of the diffraction peak of the (311) reflection in the diffusion couples annealed at 1100 °C in AMFs of 0 T and 0.1 T are 4.65 × 10^13^/m^2^ and 7.65 × 10^13^/m^2^, respectively. Thus, the broadening of XRD peaks demonstrates that the AMF results in an increase in the dislocation density in the diffusion couples.

Additionally, in order to examine whether creep deformation owing to clamping during annealing had an effect on the relative change in dislocation density with and without an AMF, a set of experiments annealing pure Ni samples after compression with and without an AMF were performed. From the FWHMs of the (111), (311) and (222) diffraction peaks, the dislocation densities in the absence of the AMF were respectively calculated to be 1.34 × 10^13^/m^2^, 4.93 × 10^13^/m^2^ and 4.03 × 10^13^/m^2^ using Dunn’s equation, whereas the dislocation densities after annealing in the AMF were 3.15 × 10^13^/m^2^, 8.60 × 10^13^/m^2^ and 5.92 × 10^13^/m^2^, respectively. The average dislocation density in the AMF increases by a factor of 1.7 in comparison with that without an AMF. It is clear that the change in the dislocation density in the deformed pure Ni sample is almost the same to that in the diffusion couples under the action of the AMF of 0.1 T. It follows that the creep deformation during annealing of the diffusion couple has no obvious effect on the relative change in dislocation density with and without the AMF.

The change of the dislocation density in the diffusion couples in the AMF can be attributed to the magnetoplastic effect (MPE). The MPE describes the change in plastic properties of materials under the action of a magnetic field^28,29^. A theoretical explanation for the MPE was proposed by Molotskii and Fleurov^30^: Strong bonds between paramagnetic obstacles, i.e. solute atoms, and dislocations usually exist only in the ground singlet (*S*) state where the electron spins are antiparallel. In the excited triplet (*T*) state with parallel spins, the bonding becomes weak or even absent. *S*-*T* transitions due to the MPE lead to weakening of bonds. Therefore, on the one hand, dislocations can depin from paramagnetic obstacles, leading to an increase in the free segment length of dislocations. The mechanism has been successfully applied to interpret principal features of the MPE^31^, the electroplastic effect^32^, multiplication of dislocations^33,34^ and work hardening^35^. Since the MPE is able to increase the dislocation density and thus provide more high diffusivity paths, it is plausible that the AMF increases the diffusivity in alloys. On the other hand, owing to the *S*-*T* transition, the solute atoms as weak obstacles become activated and thus diffuse more quickly. However, this contribution to the enhanced diffusivity is expected to be small because they only account for a fairly low fraction of total solute atoms. It is worth noting that for both the multiplication of dislocations and for activated paramagnetic obstacles, two contributions should lead to a reduction of the activation energy for diffusion. Nevertheless, the change in activation energy in the AMF was not observed in the experimental error (Table 1).

In order to substantiate the qualitative reasoning above, a more quantitative estimation of the effect of the MPE is developed. In the theory by Molotskii and Fleurov, the average free segment length of dislocations in a magnetic field is given by^30^,

1 $$L(H)=L(0)\frac{(1-p)+p{\rho }_{ss}(0)}{(1-p)+p{\rho }_{ss}(H)}$$

where *L*(*H*) and *L*(0) are the average free segment lengths with and without a magnetic field, *p* is the probability that there is a kink in the vicinity of the obstacle, *ρ*_ss_(*H*) and *ρ*_ss_(0) are the populations of the *S* state of radial pairs formed by dislocation cores and obstacle dangling bonds with and without a magnetic field, respectively. In the absence of the magnetic field, *ρ*_ss_(0) = 1/4, whereas *ρ*_ss_(*H*) can be given below

2 $${\rho }_{ss}(H)=\frac{1}{4}\frac{(1+\frac{{t}_{1}}{{\tau }_{0}})(1+\frac{{t}_{2}}{{\tau }_{0}})+\frac{{H}^{2}}{{H}_{m}^{2}}}{(1+\frac{{t}_{1}}{{\tau }_{0}})(1+\frac{{t}_{2}}{{\tau }_{0}})+(1+\frac{{\tau }_{0}}{{t}_{2}})\frac{{H}^{2}}{{H}_{m}^{2}}}$$

where *t*_1_ and *t*_2_ are the times of longitudinal and transversal spin relaxation, *τ*_0_ is the average time for the radical pairs to pass the resonance region and ${H}_{m}=\hslash /({\rm{\Delta }}g{\mu }_{B}\sqrt{{t}_{1}{t}_{2}})$, in which $\hslash$ is the Dirac constant, Δ*g* is the *g*-factor difference of the radical pair states and *μ*_*B*_ is the Bohr magneton.

In a weak magnetic field (*H*«*H*_m_), equation (1) can be reduced to

3 $$L(H)=L(0)(1+\frac{{H}^{2}}{{H}_{0}^{2}})$$

where ${H}_{0}={H}_{m}{[\frac{(1-p)+p{\rho }_{ss}(0)}{p{\rho }_{ss}(0)}(\frac{2{t}_{1}}{{\tau }_{0}})(1+\frac{{t}_{1}}{{\tau }_{0}})(1+\frac{{t}_{2}}{{\tau }_{0}})]}^{\frac{1}{2}}$ is the characteristic magnetic field inducing depinning of dislocations from paramagnetic centers. The experimental values of *H*_0_ for metals are in the order of several hundred mT^30^.

The dislocation density is directly related to the free segment length, yielding

4 $${\rho }_{dH}={\rho }_{d0}(1+\frac{{H}^{2}}{{H}_{0}^{2}})$$

where *ρ*_*dH*_ and *ρ*_*d*0_ are the dislocation densities with and without the magnetic field, respectively.

Considering the role of dislocations in bulk diffusion, one can estimate the effective diffusion coefficient according to work by Hart^36^. For the case of long diffusion annealing and high dislocation density, the effective diffusion coefficient *D*_*eff*_ is written as^36^,

5 $${D}_{eff}={D}_{l}[1+\pi {r}^{2}{\rho }_{d}(\frac{{D}_{d}}{{D}_{l}}-1)]$$

where *D*_*l*_ and *D*_*d*_ are the diffusion coefficients of lattice and dislocation, respectively, and *r* is the radius of the dislocation pipe that generally is taken as 0.5 nm for estimation. *D*_*d*_ has been measured as ${D}_{d}=2\times {10}^{-3}\exp (-18545/T)$ for dislocation pipe diffusion in Ni^37^, and *D*_*l*_ as ${D}_{l}=9.4\times {10}^{-6}\exp (-32009/T)$ for lattice diffusion^21^.

It is assumed that *D*_*l*_ and *D*_*d*_ are not affected by the external AMF. For simplicity, the relative diffusion coefficient *D*_*R*_, i.e. the ratio of the effective diffusion coefficient with an AMF to that without an AMF, is used. In a weak magnetic field the relative diffusion coefficient can be expressed as following

6 $${D}_{R}=\frac{{D}_{eff}(H)}{{D}_{eff}(0)}=1+A\cdot \frac{{H}^{2}}{{H}_{0}^{2}}$$

where $A=\frac{\pi {r}^{2}{\rho }_{d0}(\frac{{D}_{d}}{{D}_{l}}-1)}{1+\pi {r}^{2}{\rho }_{d0}(\frac{{D}_{d}}{{D}_{l}}-1)}$ is the proportionality constant that is related to the dislocation densities of two end members of the diffusion couple without an AMF, the radius of the dislocation pipe, the dislocation diffusion coefficient and the lattice diffusion coefficient.

**Figure 4:**
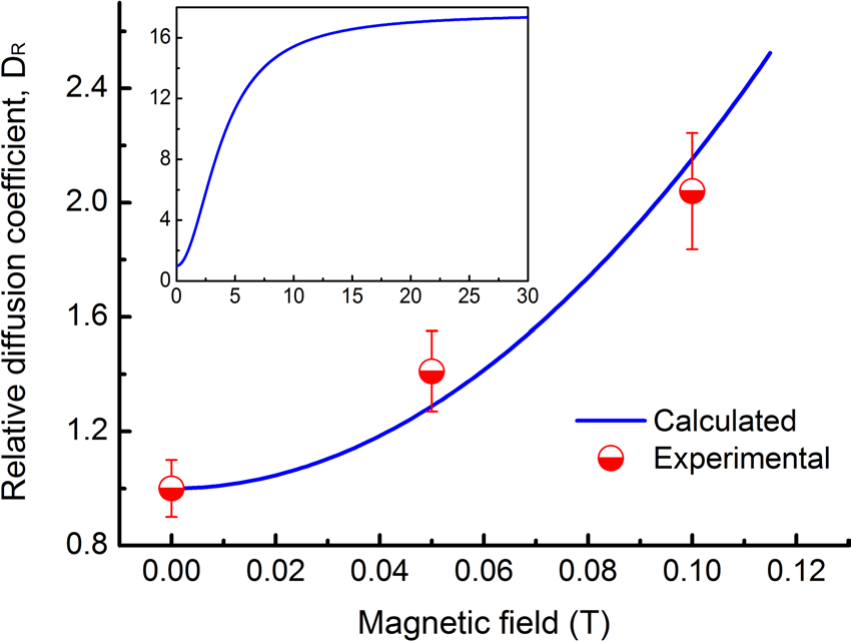
Theoretical and experimental relative diffusion coefficients at 1000 °C in the AMF. The following parameters are used: *r* = 0.5 nm, *H*_0_ = 0.093 T. The inset displays the variation of *D*_R_ with AMF intensity according to equation (1).

Equation (6) indicates a quadratic dependence of the diffusion coefficient on the magnetic field intensity in the range of weak AMFs. Figure 4 compares the theoretical and experimental relative diffusion coefficients in the AMF. The experimental data coincides with theoretical prediction. It is worth noting that at a given temperature the diffusion coefficient will deviate from the quadratic relation and gradually approach a constant value with increasing magnetic field intensity, as shown in the inset in Fig. 4. This is because that the average free segment length and the corresponding dislocation density will approach a saturated value as the AMF intensity increases (equation (1)). Experimental validation of the diffusion behavior in strong AMFs needs further investigation.

It should be pointed out that the effect of eddy current heating can be excluded to be responsible for faster diffusion. Accurate temperature measurements showed that the temperature change in the sample induced by an AMF of 0.1 T did not exceed 2 °C when the temperature of the resistance furnace was kept at 1100 °C. The slightly higher temperature accelerated the diffusivity by up to 4%, distinctly less than the observed increase in diffusivity of 105% in 0.1 T. Additionally, the effect of magnetostriction may be ruled out since the two end materials of diffusion couples are paramagnetic at annealing temperatures.

## Conclusions

In summary, the diffusivity of Cr in the Ni-Cr alloys was significantly enhanced in AMFs. The increase in diffusivity was mainly ascribed to the multiplication of dislocations due to the MPE, which provided more high-diffusivity paths. The broadening of XRD peaks demonstrates the increase in the dislocation density in the diffusion couples annealed in the AMFs. According to the change in dislocation density, a quantitative estimation of the change in diffusivity in AMFs was performed, which showed reasonable agreement between the experimental and calculated results.

## Methods

**Figure 5:**
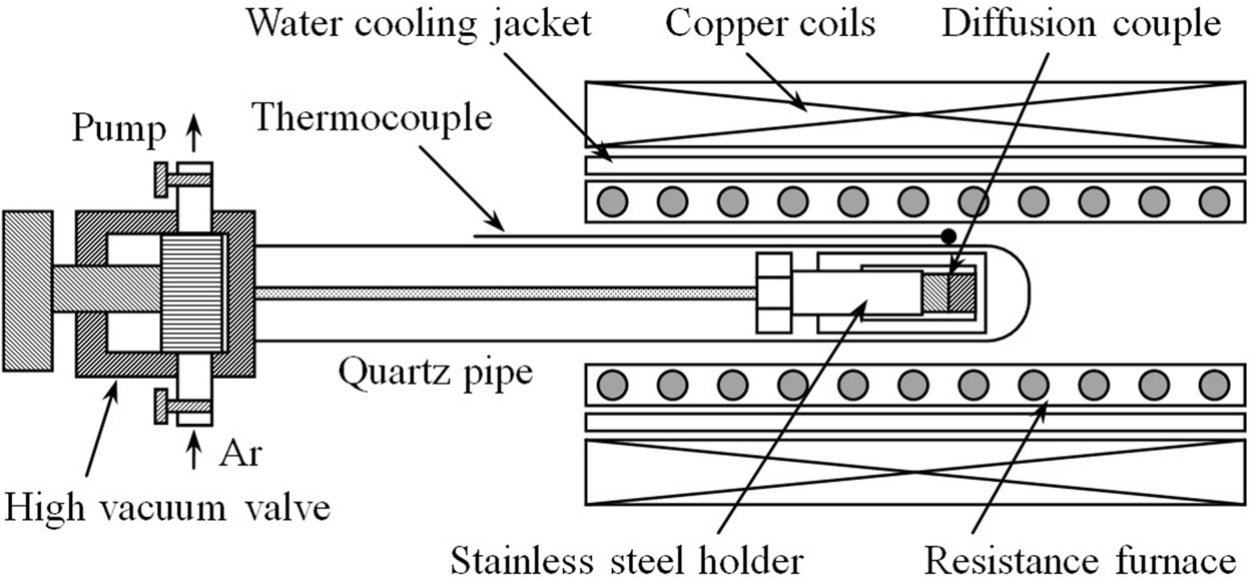
Schematic diagram of experimental apparatus for diffusion in the AMF.

The diffusion couple under investigation consisted of pure Ni (99.99%) and Ni-10wt%Cr. The Ni-10wt%Cr alloy was prepared with pure Ni and pure Cr (99.99%) using vacuum induction melting. The composition of the alloy was confirmed by EDX as Ni-10.2 wt%Cr. The alloy ingot was homogenized at 1275 °C for 32 hours. The pure Ni block was annealed for grain growth at 1300 °C for 30 hours. Heat treated ingots were cut into bars with dimensions of 4 × 4 × 3 mm that were used as the two end members of the diffusion couples. Both kinds of bars were ground using a sequence of abrasive papers up to 2000 grit and polished with 0.1μm diamond powder. Two end members were clamped using a stainless steel holder and thus formed Ni/Ni-10wt%Cr diffusion couple. The couple was transferred into a quartz tube that was closed by a high vacuum valve. Prior to the diffusion experiment, the quartz tube was purged with high purity Ar and then evacuated to 10^−3^ Pa using a turbo-molecular pump. The process was repeated five times and finally the quartz tube was again evacuated to 10^−3^ Pa. The experimental setup consisted of a resistance furnace and a surrounding AMF generator, as shown in Fig. 5. The diffusion couple was placed in the region of both maximum AMF intensity and homogeneous temperature. The temperature of the furnace was monitored by an S-type thermocouple (Pt/Pt-10Rh). An AMF with a frequency of 50 Hz was produced using copper coils that were water cooled.

The diffusion couples were annealed for 32 hours at 1000 °C, 1050 °C and 1100 °C, respectively, at different AMF intensities. One side of the annealed samples was again ground and polished. The concentration distribution perpendicular to the bonding interface was measured with Electron Probe Micro Analysis (EPMA) (EPMA-1610, Shimadzu Corporation). From concentration vs. distance profiles, interdiffusion coefficients were evaluated using the Boltzmann-Matano method.

The dislocation densities of the diffusion couples was assessed by X-Ray Diffraction (XRD) (18 kW D/MAX2500V + /PC, Rigaku Corporation) technique. The line profiles of different reflections were measured by an X-ray diffractometer at the scanning rate of 0.2°/min. From the FWHM of the diffraction peak, the dislocation density could be evaluated.

The pure Ni samples with dimension of 6 × 6 × 5 mm were compressed by 10% using a thermo-mechanical testing machine (Gleeble 3500, Dynamic Systems Inc.). Subsequently they were annealed for 24 h at 1000 °C with and without the AMF of 0.1 T without applying any mechanical stress. The dislocation densities in the pure Ni samples after annealing were analyzed using XRD.
